# Circulating biomarkers in older adults with and without sarcopenia: a systematic review and meta-analysis

**DOI:** 10.1093/gerona/glag140

**Published:** 2026-05-27

**Authors:** Konstantinos Prokopidis, Colleen S Deane, Zoubayda Baoubbou, Charlotte Beaudart

**Affiliations:** Department of Musculoskeletal Ageing and Science, Institute of Life Course and Medical Sciences, University of Liverpool, Liverpool, United Kingdom; Human Development and Health, Faculty of Medicine, University of Southampton, Southampton General Hospital, Southampton, United Kingdom; Public Health Aging Research and Epidemiology (PHARE) Group, Research Unit in Clinical Pharmacology and Toxicology (URPC), NAmur Research Institute for LIfe Sciences (NARILIS), University of Namur, Namur, Belgium; Public Health Aging Research and Epidemiology (PHARE) Group, Research Unit in Clinical Pharmacology and Toxicology (URPC), NAmur Research Institute for LIfe Sciences (NARILIS), University of Namur, Namur, Belgium; (Medical Sciences Section)

**Keywords:** Sarcopenia, Biomarkers, Ageing, GDF-15, IGF-1

## Abstract

**Background:**

Sarcopenia, the age-related loss of muscle mass and strength, poses a significant health and economic burden. This systematic review and meta-analysis evaluates circulating biomarkers (activin A, follistatin, growth differentiation factor (GDF-15), myostatin, growth hormone, insulin growth factor-1 (IGF-1), free and total testosterone) that may be associated with sarcopenia in community-dwelling older adults.

**Methods:**

Following PRISMA 2020 guidelines, we searched PubMed, Scopus, Web of Science, and Cochrane Library from inception to June 2025. Studies included adults without major comorbidities aged >60 years with sarcopenia defined by established consensus. Standardized mean differences (SMDs) were calculated using a random-effects model. Heterogeneity was assessed via *I*^2^ and meta-regressions, while Egger’s test was employed for publication bias.

**Results:**

From 3488 records, 26 observational studies were included (*n* = 1345 adults with sarcopenia, 48.3% females, mean age 67.9-88.1 years). Adults with sarcopenia showed elevated GDF-15 (*k* = 5, SMD: 0.26, 95% confidence interval (95% CI), 0.03 to 0.50, *I*^2^ = 64%, *p* = .03) and reduced IGF-1 (*k* = 11, SMD: −0.40, 95% CI, −0.54 to −0.27, *I*^2^ = 36%, *p* < .01) compared to controls without sarcopenia. No significant differences were found between groups for the other circulating biomarkers.

**Conclusions:**

Elevated circulating IGF-1 and, to a lesser extent, GDF-15, may be promising biomarkers for sarcopenia. Larger, longitudinal studies are needed to address heterogeneity and causality.

## Introduction

Sarcopenia, a condition characterized by the age-associated decline in skeletal muscle mass and strength, represents a significant public health concern.^1^ Sarcopenia is linked to increased risks of frailty, falls, disability, and mortality,^2^ affecting the quality of life and financial status of older populations.^3–7^ Globally, the prevalence of sarcopenia varies considerably, largely due to different ethnicities and lifestyles, as well as differing diagnostic criteria established by the Asian Working Group for Sarcopenia (AWGS) and the European Working Group on Sarcopenia in Older People (EWGSOP).^8–10^ For instance, such differences may be attributed to authors using a more severe or alternative definition of sarcopenia, such as using low appendicular lean mass with low handgrip strength or low lean mass with a parameter of low physical performance.^11^ Despite significant advancements in diagnostic technologies, the accurate assessment and diagnosis of sarcopenia remains a complex and time-consuming process, clinically necessitating costly equipment and highly skilled personnel. Consequently, there is a pressing socioeconomic need to discover minimally invasive biomarkers for sarcopenia. Such biomarkers would facilitate rapid and precise diagnosis, thereby enabling patients to access timely and effective interventions in order to mitigate sarcopenia onset/progression.^12^^,^^13^ Associated factors with the pathophysiology of sarcopenia may be alterations in circulating levels of several hormones and growth factors. Testosterone, insulin-like growth factor-1 (IGF-1), and growth hormone are pivotal anabolic mediators essential for the maintenance of muscle mass and function.^14^ In contrast, circulating levels of growth differentiation factor-15 (GDF-15) and activin A are key negative regulators of muscle growth,^15^^,^^16^ while the link of follistatin and myostatin may be less clear.^17–20^

Testosterone, for instance, is a key regulator of muscle protein synthesis and satellite cell function, and its decline is associated with reductions in muscle mass and strength.^21^^,^^22^ Similarly, IGF-1 and growth hormone are integral to anabolic signaling pathways that stimulate muscle hypertrophy and/or repair.^23^^,^^24^ In individuals with sarcopenia, the attenuation of these markers likely contributes to impaired muscle regeneration and accelerated muscle loss. Conversely, elevated levels of myostatin and activin A in sarcopenic individuals may inhibit myogenesis and exacerbate muscle atrophy, highlighting their role as critical catabolic mediators.^25^^,^^26^ Biomarkers such as GDF-15 and follistatin highlight further a relationship between systemic inflammation, myostatin inhibition, and regulation of muscle repair.^27^^,^^28^

Existing evidence on the associations between these biomarkers and sarcopenia remains inconsistent, owing to methodological and tissue sampling heterogeneity and limited sample sizes. By examining these biomarkers within the context of different sarcopenia definitions, this systematic review and meta-analysis aims to unravel, in part, biological signatures associated with sarcopenia.

## Methods

The revised 2020 Preferred Reporting Items for Systematic Reviews and Meta-Analyses (PRISMA) guidelines were followed throughout the conduct of this systematic review and meta-analysis.^29^ The completed PRISMA checklist is available in the Supplementary Materials. The protocol is registered in the International Prospective Register of Systematic Reviews (PROSPERO) (CRD42024556846).

### Search strategy

From inception until June 2025, four databases (PubMed, Scopus, Web of Science, and Cochrane Library) were independently searched by two investigators (K.P. and Z.B.). The employed terms used for the libraries are shown in Table S1. Furthermore, the reference lists of all included studies were thoroughly examined by hand to identify additional publications that may meet our criteria. Citations from prior systematic reviews and related review articles were manually reviewed and cross-referenced to uncover any potential further studies aligning with our inclusion criteria. Experts were also contacted for any potential missing references.

### Inclusion and exclusion criteria

Studies were included based on the following criteria: 1) baseline cross-sectional, fasted blood-based biomarker values from experimental or observational studies for groups with or without sarcopenia; 2) a mean age above 60 years in both groups (sarcopenia and non-sarcopenia); 3) consistent definitions of sarcopenia according to the EWGSOP1 or EWGSOP2, Foundation for the National Institutes of Health Sarcopenia Project (FNIH), AWGS 2014, or AWGS 2019, Society on Sarcopenia, Cachexia and Wasting Disorders (SCWD), International Working Group on Sarcopenia (IWGS), or Sarcopenia Definitions and Outcomes Consortium (SDOC); 4) healthy, community-dwelling participants.

Studies were excluded if: 1) Sarcopenia was diagnosed on the basis of a single biomarker (eg, skeletal muscle mass only); 2) Participants were pre-/post-operative hospitalized or were disease-specific (ie, osteosarcopenia, sarcopenic obesity, and cachexia); 3) Sarcopenia definitions from non-appropriate cutoffs derived by the authors’ choice that are not compatible with established consensus.

### Outcomes of interest

The section of circulating biomarkers of interest is based on the European Society for Clinical and Economic Aspects of Osteoporosis, Osteoarthritis, and Musculoskeletal Diseases (ESCEO) and the Center Académique de Recherche et d‘Expérimentation en Santé (CARES SPRL) recommendations for biomarkers to be measured in clinical trials on sarcopenia.^30^ The following myokines and hormones were investigated as outcomes in the present meta-analysis: activin A, follistatin, GDF-15, myostatin, growth hormone, IGF-1, free testosterone, and total testosterone. These markers were based on their relevance to skeletal muscle physiology, their ability to reflect the anabolic/catabolic equilibrium central to sarcopenia, and their practical utility, considering the feasibility in measuring and detecting these markers.

### Data extraction and quality assessment

Two investigators (K.P. and Z.B.) extracted data independently, including the name of the first author, publication date, country of origin, participants’ sample size, mean age, sex, and body mass index (BMI), study design, outcomes of interest, body composition assessment tool, and method of outcomes’ assessment/measurement. Disagreements were resolved by a third investigator (C.B.).

The Newcastle-Ottawa Scale (NOS) was utilized to assess the studies’ quality.^31^ NOS assigns a maximum of 9 points based on three quality parameters: selection, comparability, and outcome, and is classified as high (<5 points), moderate (5-7 points), or low (8-9 points)^32^ quality. The evaluation was made independently by two investigators (K.P. and Z.B.).

### Statistical analysis

Quantitative data were treated as continuous measurements, and differences in outcomes between those with sarcopenia vs without sarcopenia were compared to determine the standardized mean differences (SMDs) for levels of activin A, follistatin, growth hormone, and myostatin, free and total testosterone, GDF-15, and IGF-1 due to different assays employed to measure their concentrations. When studies reported median values and interquartile ranges (IQR), the formula ‘standard deviation (*SD*) = width of IQR/1.35’ was used to estimate the mean and missing *SDs*.^33^ Due to expected heterogeneity between studies, a random-effects model and the inverse-variance approach were used to determine statistical significance.

Statistical heterogeneity of outcomes across studies was measured using the overlap of their confidence intervals (95% CI) and expressed as Cochran’s Q (χ^2^ test) and *I*^2^ measurements. Low heterogeneity was defined as *I*^2^ between 30% and 49%, moderate heterogeneity between 50% and 74%, and high heterogeneity between 75% and above.^34^

In case of high heterogeneity, a random-effects meta-regression was conducted to investigate potential sources of variability that could alter estimate rates when there were 10 or more studies.^35^ The meta-regression included factors such as age, BMI, proportion of females, definition of sarcopenia, and body composition assessment tool. To assess the risk of publication bias in our analysis, used Egger’s weighted regression test and generated funnel plots for visual inspection. A leave-one-out sensitivity analysis has been performed on a significant meta-analytical model to test the robustness of the results. The meta-analysis was synthesized using Review Manager (RevMan 5.4.1) software, and a *p* value of <.05 was considered statistically significant.

## Results

**Figure 1:**
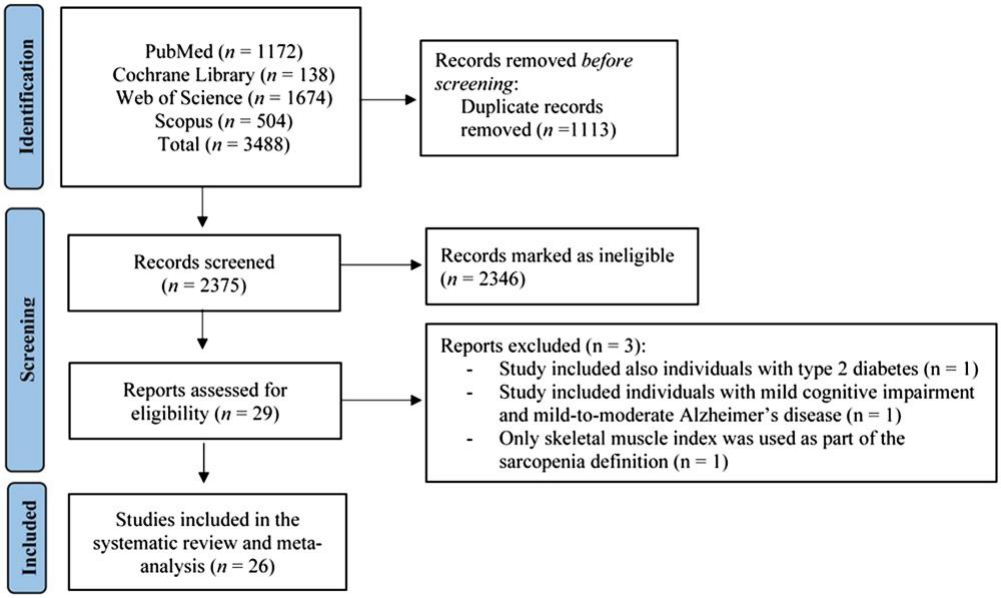
PRISMA flowchart.

From an initial pool of 3488 full texts, 1113 were removed as duplicates. Following a record screening of titles and abstracts, 29 studies were assessed for eligibility. From these, one study included adults with type 2 diabetes,^36^ one study included adults with mild cognitive impairment or mild-to-moderate Alzheimer’s disease,^37^ and one study used skeletal muscle index as a sole component of sarcopenia definition.^38^ Finally, 26 observational studies were included in the systematic review and meta-analysis^18^^,^^19^^,^^39–62^ (Figure 1). From the available data in those with sarcopenia, the minimum mean age of the included cohorts was 67.9 years, while the maximum mean age was 88.1 years. Mean BMI ranged from 19.8 kg/m^2^ to 28.5 kg/m^2^. Of the 1345 adults with sarcopenia, about half were females. Additionally, 18 cohorts used dual x-ray absorptiometry (DXA), seven used bioelectrical impedance (BIA), while one did not use any of these tools, given that calf circumference (CC) was applied as part of the sarcopenia definition. Characteristics of the included studies are presented in Table 1.

**Table 1:** Characteristics of the included studies.

Author (year)	Country	Biomarkers	Sarcopenia diagnosis			Sarcopenia			No sarcopenia		
			Sarcopenia definition	Sarcopenia instruments—Muscle mass Muscle strength Physical performance (optional)	Tool for body composition	Sample size (M/F)	Age (years)	BMI (kg/m^2^)	Sample size (M/F)	Age (years)	BMI (kg/m^2^)
**Kuo et al. 2019**	Taiwan	Testosterone, growth hormone	AWGS2014	SMI: (7.0 kg/m^2^ for men and 5.4 kg/m^2^ for women; handgrip strength (<26 kg for men and <18 kg for women)	DXA	50 (36/14)	76.7 ± 5.3	22.3 ± 3.2	681 (350/331)	73.1 ± 5.4	24.7 ± 3.1
**Shin et al. 2023**	South Korea	Testosterone, free testosterone, myostatin, GDF-15	AWGS2019	SMI: (7.0 kg/m^2^ for men and 5.4 kg/m^2^ for women; handgrip strength (<26 kg for men and <18 kg for women); gait speed of <1.0 m/s, total SPPB score of ≤9, or 5CST time of ≥12 s	DXA	176 (102/74)	77.7 ± 3.8	22.2 ± 2.8	845 (396/449)	75.3 ± 3.7	24.6 ± 2.8
**Liang et al. 2023 (AWGS2014)**	Taiwan	Testosterone, IGF-1	AWGS 2014	SMI: (7.0 kg/m^2^ for men and 5.4 kg/m^2^ for women; handgrip strength (<26 kg for men and <18 kg for women); gait speed of <0.8 m/s	DXA	50 (36/14)	76.7 ± 5.3	21.6 ± 2.7	681 (350/331)	73.1 ± 5.4	24.9 ± 3.5
**Liang et al. 2023 (AWGS2019)**	Taiwan	Testosterone, IGF-1	AWGS 2019	SMI: (7.0 kg/m^2^ for men and 5.4 kg/m^2^ for women; handgrip strength (<28 kg for men and <18 kg for women); gait speed of <1.0 m/s	DXA	62 (47/15)	76.9 ± 5.2	21.7 ± 2.6	669 (339/330)	73.1 ± 5.3	25.0 ± 3.5
**Seo et al. 2020**	South Korea	Myostatin, activin A, GDF-15, follistatin	EWGSOP2 and IWGS	SMI <5.67 kg/m^2^; handgrip strength <20 kg; gait speed of <1.0 m/s	DXA	27 (0/27)	71.4 ± 4.9	22.5 ± 1.8	32 (0/32)	71.6 ± 4.1	23.6 ± 2.3
**Miyamoto et al. 2021**	Japan	IGF-1	AWGS2014	SMI: (7.0 kg/m^2^ for men and 5.4 kg/m^2^ for women; handgrip strength (<26 kg for men and <18 kg for women)	DXA	39 (14/25)	75.9 ± 5.7	21.1 ± 2.4	453 (158/295)	64.9 ± 10.5	23.4 ± 3.0
**Ke et al. 2022**	China	Testosterone	EWGSOP2	CC <31 cm; handgrip strength <27 kg; gait speed of ≤0.8 m/s	–	64 (64/0)	88.1 ± 4.3	–	296 (296/0)	85.8 ± 4.0	–
**Lu et al. 2022**	Singapore	IGF-1	AWGS2019	SMI: (7.0 kg/m^2^ for men and 5.4 kg/m^2^ for women; knee extension strength (≤18 kg for men and ≤16 kg for women); gait speed of <1.0 m/s	DXA	60 (24/36)	70.0 ± 4.7	21.5 ± 2.7	9 (6/3)	69.9 ± 4.0	23.7 ± 2.8
**Ferrari et al. 2021**	Germany	IGF-1, growth hormone	EWGSOP2	SMI: (7.0 kg/m^2^ for men and 5.5 kg/m^2^ for women; handgrip strength (<27 kg for men and <16 kg for women)	DXA	35 (18/17)	84.0 ± 8.1	22.8 ± 2.3	96 (32/64)	83.0 ± 7.6	26.3 ± 5.4
**Du et al. 2021**	China	Myostatin, follistatin	AWGS2014	SMI: (5.4 kg/m^2^ for women; handgrip strength (<18 kg for women); gait speed of <0.8 m/s	DXA	51 (0/51)	69.5 ± 10.7	20.3 ± 1.9	193 (0/193)	64.5 ± 8.3	25.1 ± 2.6
**Nga et al. 2021**	South Korea	GDF-15	AWGS2019	SMI: (7.0 kg/m^2^ for men and 5.7 kg/m^2^ for women; handgrip strength (≤28 kg for men and ≤18 kg for women); gait speed of <1.0 m/s, total SPPB score of ≤9, or 5CST time of ≥12 s	BIA	21 (7/14)	71.9 ± 4.7	24.0 ± 3.3	104 (50/54)	68.6 ± 6.5	26.5 ± 3.1
**Choi et al. 2024**	South Korea	Free testosterone	AWGS2019	SMI: (7.0 kg/m^2^ for men; handgrip strength (<28 kg for men); total SPPB score of ≤9	DXA	90 (90/0)	70+	–	821 (821/0)	70+	–
**Kim et al. 2022**	South Korea	GDF-15	AWGS2019	SMI: (7.0 kg/m^2^ for men and 5.7 kg/m^2^ for women; handgrip strength (≤28 kg for men and ≤18 kg for women); gait speed of <1.0 m/s, total SPPB score of ≤9, or 5CST time of ≥12 s	DXA	–	65+	–	775 -	65+	–
**Hata et al. 2023**	Japan	IGF-1	AWGS2019	SMI: (<7.0 kg/m^2^ for men and <5.7 kg/m^2^ for women; handgrip strength (<28 kg for men and <18 kg for women); gait speed of <1.0 m/s	BIA	105 (44/61)	86.9 ± 1.4	20.7 ± 3.1	890 (453/437)	86.5 ± 1.4	23.6 ± 2.9
**Yen et al. 2022**	Taiwan	Myostatin	AWGS2019	SMI: (<7.0 kg/m^2^ for men and <5.4 kg/m^2^ for women; handgrip strength (<28 kg for men and <18 kg for women); gait speed of <1.0 m/s	DXA	46 (19/27)	74.8 ± 7.4	19.8 ± 2.4	53 (7/46)	72.0 ± 8.4	23.1 ± 2.9
**Kirk et al. 2020**	Australia	Testosterone	EWGSOP2	SMI: (7.0 kg/m^2^ for men and 5.5 kg/m^2^ for women; handgrip strength (<27 kg for men and <16 kg for women); gait speed ≤0.8 m/s	DXA	32 (11/21)	80.3 ± 7	22.9 ± 3.6	397 (123/274)	75 ± 8.2	27.5 ± 5
**Hofmann et al. 2015**	Austria	IGF-1, Myostatin, activin A, GDF-15, follistatin	EWGSOP1	SMI: (≤6.75 kg/m^2^ for women); peak torque of knee extension at 60°/s < 61.5 Nm; gait speed of ≤1.0 m/s	DXA	9 (0/9)	83.4 ± 3.8	24.4 ± 3.5	78 (0/78)	81.7 ± 6.2	29.6 ± 4.3
**Xu et al. 2022**	China	IGF-1, growth hormone	AWGS2019	SMI: (<7.0 kg/m^2^ for men and <5.4 kg/m^2^ for women; handgrip strength (<28 kg for men and <18 kg for women); gait speed of ≤1.0 m/s, or 5CST time of ≥12 s	DXA	57 (34/23)	75.7 ± 8.4	21.5 ± 4.3	64 (23/41)	65.7 ± 9.1	24.1 ± 3
**Volpato et al. 2014**	Italy	Testosterone, IGF-1	EWGSOP1	SMI: (7.0 kg/m^2^ for men and 5.5 kg/m^2^ for women; handgrip strength (<30 kg for men and <20 kg for women); gait speed <0.8 m/s	BIA	55 (19/36)	83.8 ± 5.9	25.4 ± 4.0	373 (184/189)	75.9 ± 4.9	28.4 ± 3.6
**Tay et al. 2015**	Singapore	Testosterone, IGF-1, myostatin	AWGS2014	SMI: (7.0 kg/m^2^ for men and 5.4 kg/m^2^ for women; handgrip strength (<26 kg for men and <18 kg for women); gait speed of <0.8 m/s	DXA	50 (16/34)	72.0 ± 8.1	21.7 ± 2.4	150 (47/103)	66.6 ± 7.3	24.7 ± 3.8
**Diago-Galmés et al. 2021**	Spain	Testosterone	EWGSOP2	ASMM: (<20 kg for men and 15 kg for women; handgrip strength (<27 kg for men and <16 kg for women); gait speed <0.8 m/s	BIA	47 (8/39)	–	–	143 (27/116)	–	–
**Yee et al. 2020**	Australia	Free testosterone, IGF-1, growth hormone	EWGSOP1 + EWGSOP2	EWGSOP1: Skeletal muscle mass index by BIA, adjusted for height <6.75 kg/m^2^ and grip strength <20 kg; EWGSOP2: Skeletal muscle mass index by BIA, adjusted for height <6.75 kg/m^2^ and grip strength <16 kg	DXA	11 (0/11)	60+	–	28 (0/28)	60+	–
**Li et al. 2019**	China	IGF-1	AWGS2014	SMI: (7.0 kg/m^2^ for men and 5.4 kg/m^2^ for women; handgrip strength (<26 kg for men and <18 kg for women); gait sped of <0.8 m/s	DXA	56 (32/24)	72.1 ± 6.5	21.4 ± 3.1	56 (30/26)	65.2 ± 4.1	24.7 ± 3.0
**Aryana et al. 2019**	Indonesia	Myostatin	AWGS2014	SMI: (7.0 kg/m^2^ for men and 5.7 kg/m^2^ for women; handgrip strength (<26 kg for men and <18 kg for women); gait speed of <0.8 m/s	BIA	45 (20/25)	60+	–	25 (15/10)	60+	–
** Echeverria et al. 2021**	Spain	Myostatin, follistatin	EWGSOP2	SMI: (7.0 kg/m^2^ for men and 5.5 kg/m^2^ for women; handgrip strength (<27 kg for men and <16 kg for women); 5CST >15 s	DXA	15 (7/8)	84.5 ± 5.1	25.2 ± 3.9	59 (24/35)	81.4 ± 5.8	29.9 ± 4.7; *n* = 58
**de Luis et al. 2024**	Spain	Myostatin	EWGSOP2	SMI: (7.0 kg/m^2^ for men and 5.5 kg/m^2^ for women; handgrip strength (<27 kg for men and <16 kg for women)	BIA	44 (20/24)	67.9 ± 2.4	21.7 ± 2.1	64 (24/40)	67.3 ± 3.1	22.8 ± 2.6
**Surmeli et al. 2022**	Turkey	Testosterone	EWGSOP2	SMI: (9.2 kg/m^2^ for men; handgrip strength (<32 kg for men)	BIA	48 (48/0)	74.3 ± 8.2	28.5 ± 3.8	145 (145/0)	70.3 ± 6.7	24.2 ± 4.0

Abbreviations: 5CST, 5-time chair stand test; ASMM, appendicular skeletal muscle mass; BIA, bioelectrical impedance; CC, calf circumference; DXA, dual x-ray absorptiometry; SMI, skeletal muscle index; SPPB, short physical performance batter.

### Biomarker differences between older adults with sarcopenia vs without sarcopenia

**Figure 2:**
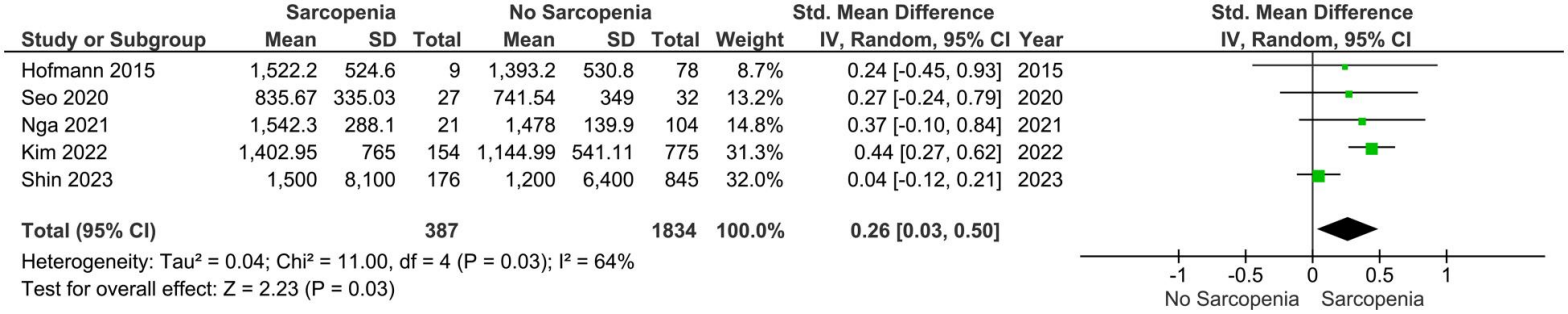
Differences in GDF-15 between adults with vs without sarcopenia.

**Figure 3:**
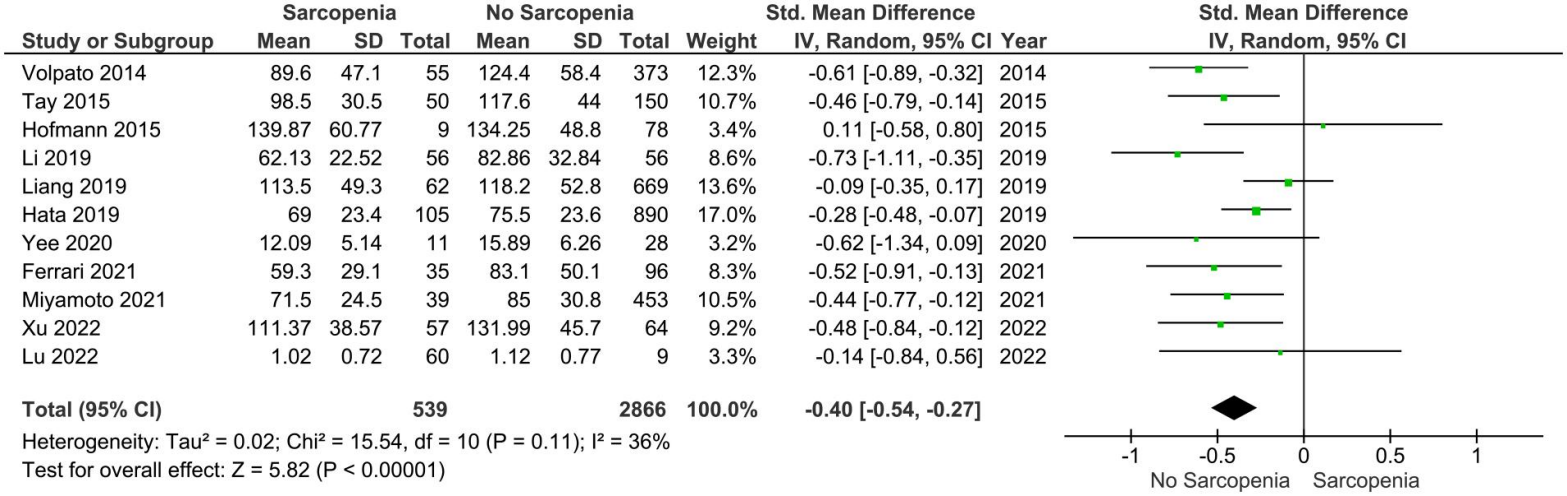
Differences in IGF-1 between adults with vs without sarcopenia.

A statistically significant difference was found pertinent to GDF-15 and IGF-1 concentrations, for which, those with sarcopenia exhibited elevated levels vs those without sarcopenia (GDF-15 → *k* = 5; SMD: 0.26, 95% CI, 0.03 to 0.50, *I*^2^ = 64%, *p* = .03; Figure 2—IGF-1 → *k* = 11; SMD: −0.40, 95% CI, −0.54 to −0.27, *I*^2^ = 36%, *p* < .01; Figure 3). Leave-one-out analyses showed unaltered results for IGF-1; however, omission of Seo et al. (2020), Nga et al. (2021), or Kim et al. (2022) showed no statistically significant findings pertaining to GDF-15 between groups (Supplementary File).

Our main analysis showed no significant differences in relation to circulating activin A levels (*k* = 2; SMD: −0.04, 95% CI, −0.77 to 0.70, *I*^2^ = 66%, *p* = .92) (Figure S1), although the analysis was based on two studies with high heterogeneity. Similarly, no statistical differences were observed regarding follistatin levels (*k* = 4; SMD: 0.30, 95% CI, −0.45 to 1.05, *I*^2^ = 89%, *p* = .43) (Figure S2). In addition, we found no differences on myostatin (*k* = 8; SMD: −0.18, 95% CI, −0.45 to 0.09, *I*^2^ = 77%, *p* = .19) (Figure S3) nor growth hormone levels between the two groups (*k* = 4; SMD: 0.13, 95% CI, −0.08 to 0.34, *I*^2^ = 16%, *p* = .21) (Figure S4). Finally, no statistically significant differences between the groups were revealed pertinent to free (*k* = 4; SMD: −0.20, 95% CI, −0.50 to 0.11, *I*^2^ = 79%, *p* = .20) (Figure S5) or total testosterone (*k* = 6; SMD: −0.04, 95% CI, −0.32 to 0.25, *I*^2^ = 82%, *p* = .79) (Figure S6).

### Publication bias and meta-regression

We performed publication bias assessment and meta-regressions based on studies that included IGF-1, considering it was the only outcome with at least 10 or more studies. We found no publication bias (*p* = .69, *t* = −0.42, *b* = −0.31, 95% CI, −0.74 to −0.12) (Table S2). Meta-regressions revealed that neither age, BMI, proportion of females, sarcopenia definition, nor body composition assessment tool influenced the results (Table S3).

### Risk of bias assessment

Our risk of bias assessment revealed no studies with a very low quality. Three studies had a score of “5,”^42^^,^^55^^,^^60^ primarily due to their statistical analysis being deemed inappropriate, poorly described or incomplete, their sample size not justified, and/or having an unsatisfactory response rate. The majority of studies had a high score, indicating overall a good quality. Details are presented in Table S4.

## Discussion

Challenges in relation to sarcopenia diagnosis have led to a need for rapid and minimally invasive biomarkers. This systematic review and meta-analysis highlights significant differences in circulating GDF-15 and IGF-1 in older adults with sarcopenia compared to those without sarcopenia, while revealing no statistically significant differences regarding activin A, follistatin, myostatin, growth hormone, or testosterone levels.

The elevated GDF-15 levels in sarcopenic adults (vs non-sarcopenic) align with its role as a stress-induced cytokine linked to muscle wasting and inflammation, which is consistent with prior studies tying GDF-15 to frailty and sarcopenia in humans.^63^^,^^64^ Elevated plasma or serum levels of GDF-15 have been demonstrated in conditions such as lung cancer, following surgery, mitochondrial disease, and advanced fibrosis,^65–68^ while stress-induced responses enhancing GDF-15 have also been found in skeletal muscle from patients admitted in intensive care units.^69^ However, during healthy states, previous work has suggested that skeletal muscle GDF-15 is linked to increased muscle strength and lower interleukin-6 levels as opposed to its circulating levels.^70^ In community-dwelling older adults with sarcopenia, these findings suggest that although circulating levels may be elevated compared to those without sarcopenia, lower values may be skeletal muscle-specific. While the cross-sectional findings of this study may be a promising marker for sarcopenia, the role of GDF-15 is complex and particularly varies by comorbidities and their severity, which are highly prevalent in advancing ages that could overlap with sarcopenia. Additionally, our leave-one-out analyses showed that results pertaining to GDF-15 between those with vs without sarcopenia are not robust, given that effect sizes and statistical significance were inconsistent. Therefore, more studies are required to examine GDF-15-based differences in these groups. Nevertheless, it is worth noting that, in this systematic review and meta-analysis, we attempted to reduce the likelihood of major comorbidities by including only community-dwelling populations.

Similarly, the lower IGF-1 in sarcopenia, with relatively low heterogeneity (*I*^2^ = 36%), reinforces its anabolic role in skeletal muscle maintenance and repair.^24^^,^^71^ IGF-1 is a primary signal of growth in response to anabolic stimulus, such as resistance exercise and nutrition, and is implicated in several pathways, including Akt activation and phosphorylation of the mammalian target of rapamycin (mTOR); critical in muscle protein synthetic processes.^72^^,^^73^ The anabolic potential of IGF-1 may also be repressed under conditions of inactivity,^74^ which is a common feature in those with sarcopenia, either as a contributor to it or a consequence of it. Research exploring the magnitude of difference at the skeletal muscle level in community-dwelling groups would help validate our findings.

Moreover, the lack of differences in myostatin and activin A challenges their presumed inhibitory roles in sarcopenia, at least from a serum level perspective, although the observed high heterogeneity (*I*^2^ = 79% and 89%, respectively) and limited study numbers (*k* = 8 and *k* = 2, respectively) limit confidence in those results. Higher mRNA myostatin expression levels have been shown in the vastus lateralis of middle-aged-to-older adults with sarcopenia and overweight or obesity vs their counterparts without sarcopenia.^26^ Similarly, higher myostatin expression has also been shown in older vs younger adults, which may be sex-specific.^75^^,^^76^ Pertaining to our findings, previous work has also demonstrated an increase in muscle mRNA myostatin content in adults with type 2 diabetes vs healthy controls, albeit no differences were depicted regarding plasma levels.^77^ In addition, follistatin, growth hormone, and testosterone also showed no significant changes between those with vs without sarcopenia, with heterogeneity ranging from minimal (*I*^2^ = 5% for growth hormone) to substantial (*I*^2^ = 82% for total testosterone), hinting at methodological or population differences (eg, sex or age) that were not captured in meta-regressions for IGF-1. The absence of publication bias and non-significant moderators, including age, BMI, or sex for IGF-1, may suggest precision; however, the limited number of studies related to other biomarkers limits generalizability. In this context, it is also worth noting that circulating levels of activin A and follistatin may also be prone to changes due to bodyweight differences,^78^^,^^79^ considering that adults without sarcopenia had generally a higher BMI than those with sarcopenia. These findings suggest that the circulating biomarker profile in sarcopenia is selective, with GDF-15 and IGF-1 emerging as key surrogate markers, potentially reflecting inflammatory and anabolic pathways, respectively. The minimal results elsewhere may signal underpowered analyses or context-specific effects (eg, disease states such as heart failure or cancer), urging caution in dismissing these circulating markers. Added to this, this variability may stem from context-specific factors, including age, sex, (non-reported) comorbidities, definition or stage of sarcopenia, which could mask consistent patterns in serum levels. For instance, myostatin may not serve as a reliable standalone circulating biomarker but could still provide valuable insights when evaluated in conjunction with other sarcopenia markers, such as muscle strength and/or physical function. This highlights the need for a multi-marker approach in sarcopenia research, where multiple biomarkers are assessed alongside clinical outcomes. Future research should prioritize larger, longitudinal cohorts to clarify causality tailored to investigating molecular pathways pertaining to sarcopenia.^80^

### Strengths and limitations

In this meta-analysis, the inclusion of eleven studies for IGF-1, alongside a low risk of bias in most studies (no overall high-risk ratings), enhances reliability, while the absence of publication bias for IGF-1 and the exploration of meta-regression moderators (age, BMI, sex, and sarcopenia definition) add further robustness. However, this study has several limitations. High heterogeneity was displayed in most outcomes (eg, *I*^2^ = 93% for follistatin, 89% for activin A), likely driven by the limited study numbers, various sarcopenia definitions, diverse populations, and different assessment methods. For instance, the paucity of studies for activin A (*k* = 2) restricts statistical power, while the inclusion of three studies with poor quality scores, due to inadequate statistical reporting or sample size justification, introduces potential bias. In addition, the cross-sectional nature of the data precludes causal inferences, whereby unexamined confounders (eg, physical activity, (non-reported) comorbidities, medication use, and dietary intake) may obscure true effect sizes that may precede or follow sarcopenia onset. Finally, the lack of sex-stratified analyses limits insight into sex-specific circulating biomarker profiles.

## Conclusions

This systematic review and meta-analysis showed that older adults with sarcopenia exhibit elevated circulating GDF-15 and reduced IGF-1 compared to those without sarcopenia. No significant differences were revealed for activin A, follistatin, myostatin, growth hormone, or testosterone, although heterogeneity and small sample sizes caution against firm conclusions. These findings position GDF-15 and IGF-1 as potentially promising blood biomarkers for sarcopenia, with potential diagnostic and therapeutic implications. Larger, longitudinal studies are needed to resolve heterogeneity and account for comorbidity status, to enhance causal effects and provide more reliable insights using multiple biomarkers.

## Supplementary Material

glag140_Supplementary_Data

## Data Availability

Data are available upon request.
