# Supplementary material for: Circulating biomarkers in older adults with and without sarcopenia: a systematic review and meta-analysis
Source: J Gerontol A Biol Sci Med Sci. 2026 May 27;81(7):glag140. doi: 10.1093/gerona/glag140 (PMC13283486; doi:10.1093/gerona/glag140)
Supplement: glag140_Supplementary_Data [file glag140_supplementary_data.zip › Supplementary File.pdf]

Supplementary File

Supplementary Figures

Figure S1. Differences in activin A between adults with vs. without sarcopenia.

	Sarcopenia			No Sarcopenia				Std. Mean Difference	
Study or Subgroup	Mean	SD	Total	Mean	SD	Total	Weight	IV, Random, 95% CI	Year
Hofmann 2015	360	370	9	870	1,170	78	45.1%	-0.45 [-1.14, 0.24]	2015
Seo 2020	380.17	90.23	27	351.26	97.45	32	54.9%	0.30 [-0.21, 0.82]	2020
**Total (95% CI)**			**36**			**110**	**100.0%**	**-0.04 [-0.77, 0.70]**	

Heterogeneity: Tau² = 0.19; Chi² = 2.93, df = 1 (P = 0.09); I² = 66%
Test for overall effect: Z = 0.10 (P = 0.92)

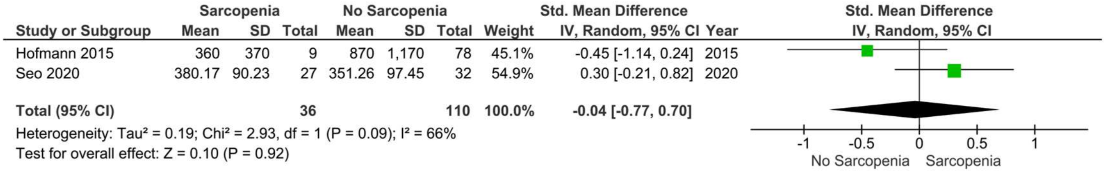

Figure S2. Differences in follistatin between adults with vs. without sarcopenia.

	Sarcopenia			No Sarcopenia				Std. Mean Difference	
Study or Subgroup	Mean	SD	Total	Mean	SD	Total	Weight	IV, Random, 95% CI	Year
Hofmann 2015	1.98	0.52	9	2.12	0.52	78	23.0%	-0.27 [-0.96, 0.42]	2015
Seo 2020	2.09	0.56	27	2.2	0.59	32	25.2%	-0.19 [-0.70, 0.33]	2020
Du 2021	18.97	6.1	51	13	4.9	193	27.2%	1.15 [0.83, 1.48]	2021
Echeverria 2021	3.19	1.23	15	2.68	1.28	59	24.6%	0.40 [-0.17, 0.97]	2021
**Total (95% CI)**			**102**			**362**	**100.0%**	**0.30 [-0.45, 1.05]**	

Heterogeneity: Tau² = 0.51; Chi² = 26.59, df = 3 (P < 0.00001); I² = 89%
Test for overall effect: Z = 0.79 (P = 0.43)

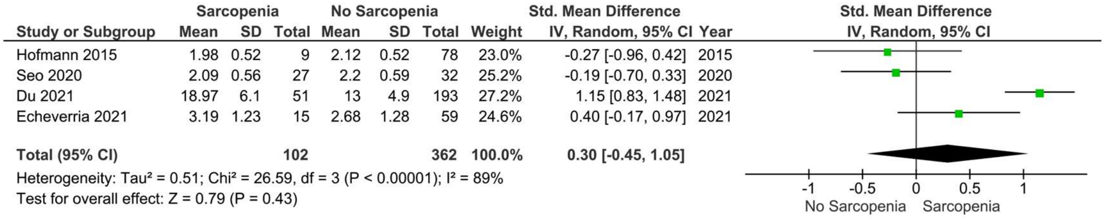

Figure S3. Differences in myostatin between adults with vs. without sarcopenia.

	Sarcopenia			No Sarcopenia				Std. Mean Difference	
Study or Subgroup	Mean	SD	Total	Mean	SD	Total	Weight	IV, Random, 95% CI	Year
Hofmann 2015	2.43	1.41	9	3.12	2.14	78	8.2%	-0.33 [-1.02, 0.36]	2015
Tay 2015	28.6	8.4	50	27.5	7.6	150	14.2%	0.14 [-0.18, 0.46]	2015
Seo 2020	2.07	0.73	27	2.06	0.87	32	10.9%	0.01 [-0.50, 0.52]	2020
Du 2021	2.75	1.6	51	3.99	1.5	193	14.3%	-0.81 [-1.13, -0.50]	2021
Echeverria 2021	2.32	0.5	15	3.61	2.03	59	9.8%	-0.70 [-1.27, -0.12]	2021
Yen 2022	9.5	4.7	46	8.5	3.9	53	12.9%	0.23 [-0.17, 0.63]	2022
Shin 2023	3.2	1	176	3.5	1.2	845	16.7%	-0.26 [-0.42, -0.09]	2023
de Luis 2024	5.01	1.87	44	4.64	2.17	64	13.1%	0.18 [-0.21, 0.56]	2024
**Total (95% CI)**			**418**			**1474**	**100.0%**	**-0.18 [-0.45, 0.09]**	

Heterogeneity: Tau² = 0.11; Chi² = 31.02, df = 7 (P < 0.0001); I² = 77%
Test for overall effect: Z = 1.30 (P = 0.19)

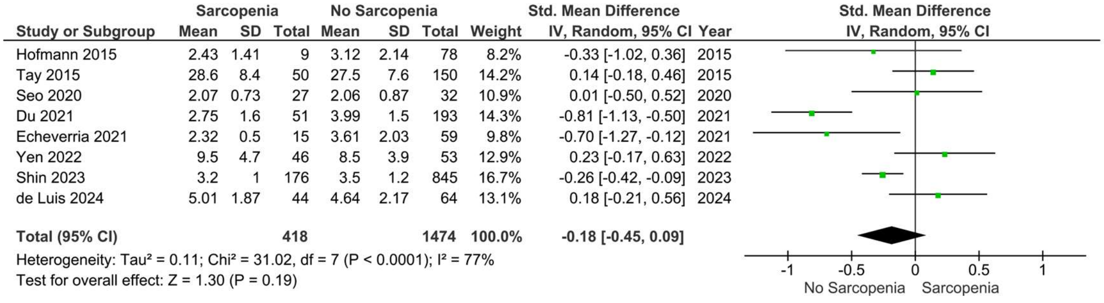

Figure S4. Differences in growth hormone between adults with vs. without sarcopenia.

	Sarcopenia			No Sarcopenia				Std. Mean Difference	
Study or Subgroup	Mean	SD	Total	Mean	SD	Total	Weight	IV, Random, 95% CI	Year
Kuo 2018	0.47	0.66	50	0.35	0.45	681	39.3%	0.26 [-0.03, 0.54]	2018
Yee 2020	1.32	1.58	11	2.52	3	28	8.3%	-0.44 [-1.14, 0.27]	2020
Ferrari 2021	1.4	1.3	35	1.3	2.4	96	24.5%	0.05 [-0.34, 0.43]	2021
Xu 2022	0.3876	0.7083	57	0.2714	0.3888	64	27.9%	0.21 [-0.15, 0.56]	2022
**Total (95% CI)**			**153**			**869**	**100.0%**	**0.13 [-0.08, 0.34]**	

Heterogeneity: Tau² = 0.01; Chi² = 3.56, df = 3 (P = 0.31); I² = 16%
Test for overall effect: Z = 1.25 (P = 0.21)

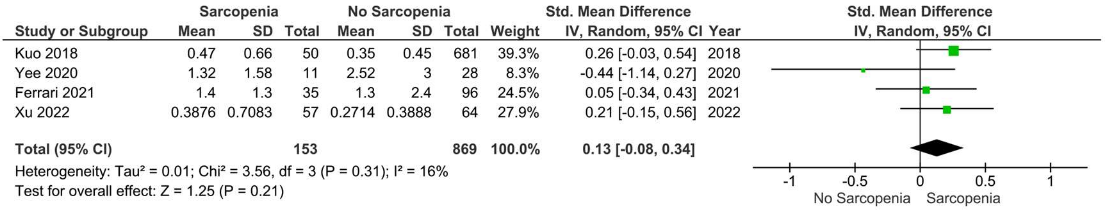

Figure S5. Differences in free testosterone between adults with vs. without sarcopenia.

	Sarcopenia			No Sarcopenia				Std. Mean Difference	
Study or Subgroup	Mean	SD	Total	Mean	SD	Total	Weight	IV, Random, 95% CI	Year
Tay 2015	0.02302	0.0071	50	0.02599	0.00647	150	25.4%	-0.45 [-0.77, -0.12]	2015
Yee 2020	5.6	1.7	11	7.08	3.67	28	12.2%	-0.45 [-1.15, 0.26]	2020
Shin 2023	0.0191	0.0173	176	0.017	0.0166	845	32.3%	0.13 [-0.04, 0.29]	2023
Choi 2024	0.03219	0.01442	90	0.03482	0.01096	821	30.1%	-0.23 [-0.45, -0.01]	2024
**Total (95% CI)**			**327**			**1844**	**100.0%**	**-0.20 [-0.50, 0.11]**	

Heterogeneity: Tau² = 0.07; Chi² = 13.96, df = 3 (P = 0.003); I² = 79%
Test for overall effect: Z = 1.27 (P = 0.20)

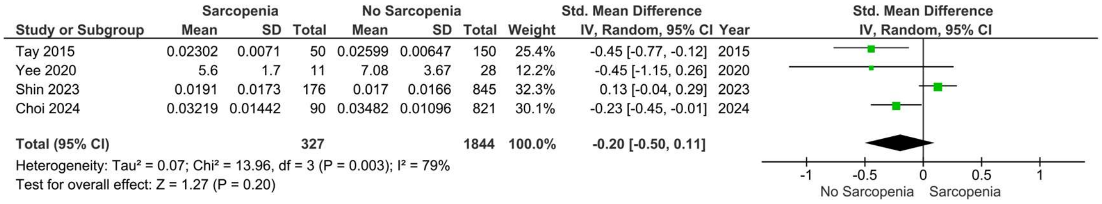

Figure S6. Differences in total testosterone between adults with vs. without sarcopenia.

	Sarcopenia			No Sarcopenia				Std. Mean Difference	
Study or Subgroup	Mean	SD	Total	Mean	SD	Total	Weight	IV, Random, 95% CI	Year
Volpato 2014	0.193	0.301	55	0.529	0.789	373	17.0%	-0.45 [-0.73, -0.17]	2014
Kuo 2018	4.712	2.295	50	4.859	1.771	681	16.9%	-0.08 [-0.37, 0.21]	2018
Liang 2019	3.74	2.564	62	2.657	2.699	669	17.4%	0.40 [0.14, 0.66]	2019
Kirk 2020	2.67	3.4	32	3.57	1.81	397	15.4%	-0.46 [-0.82, -0.09]	2020
Ke 2022	4.12	2.22	64	3.74	1.81	296	17.2%	0.20 [-0.07, 0.47]	2022
Surmeli 2022	3.33	1.154	48	3.22	0.996	145	16.1%	0.11 [-0.22, 0.43]	2022
**Total (95% CI)**			**311**			**2561**	**100.0%**	**-0.04 [-0.32, 0.25]**	

Heterogeneity: Tau² = 0.10; Chi² = 27.67, df = 5 (P < 0.0001); I² = 82%
Test for overall effect: Z = 0.26 (P = 0.79)

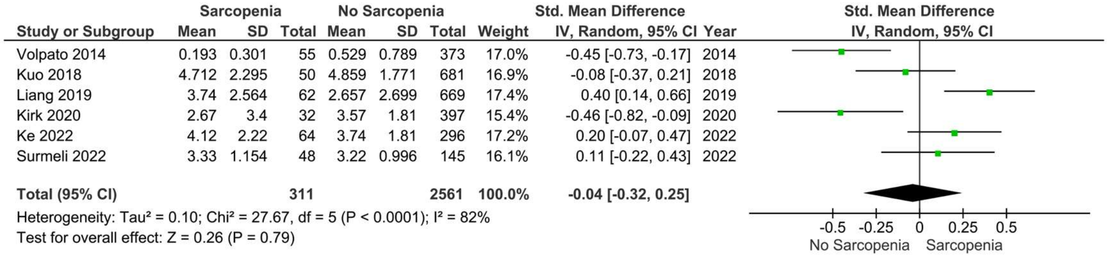

Leave-one-out sensitivity analyses

**IGF-1**	
Volpato 2014 – out	SMD: -0.37, 95%CI: -0.51 – -0.23, I^2 = 30%, P < 0.01
Tay 2015 – out	SMD: -0.39, 95%CI: -0.55 – -0.24, I^2 = 41%, P < 0.01
Hofmann 2015 – out	SMD: -0.42, 95%CI: -0.55 – -0.28, I^2 = 33%, P < 0.01
Li 2019 – out	SMD: -0.37, 95%CI: -0.50 – -0.24, I^2 = 26%, P < 0.01
Liang 2019 – out	SMD: -0.44, 95%CI: -0.56 – -0.32, I^2 = 8%, P < 0.01
Hata 2019 – out	SMD: -0.43, 95%CI: -0.58 – -0.27, I^2 = 36%, P < 0.01
Yee 2020 – out	SMD: -0.39, 95%CI: -0.53 – -0.25, I^2 = 40%, P < 0.01
Ferrari 2021 – out	SMD: -0.39, 95%CI: -0.54 – -0.24, I^2 = 40%, P < 0.01
Miyamoto 2021 – out	SMD: -0.40, 95%CI: -0.55 – -0.25, I^2 = 42%, P < 0.01
Xu 2022 – out	SMD: -0.39, 95%CI: -0.54 – -0.25, I^2 = 41%, P < 0.01
Lu 2022 – out	SMD: -0.41, 95%CI: -0.55 – -0.27, I^2 = 40%, P < 0.01
**GDF-15**	
Hofmann 2015 – out	SMD: 0.27, 95%CI: 0.01 – 0.53, I^2 = 73%, P = 0.04
Seo 2020 – out	SMD: 0.26, 95%CI: -0.01 – 0.54, I^2 = 73%, P = 0.06
Nga 2021 – out	SMD: 0.25, 95%CI: -0.03 – 0.52, I^2 = 72%, P = 0.08
Kim 2022 – out	SMD: 0.10, 95%CI: -0.04 – 0.25, I^2 = 0%, P = 0.17
Shin 2023 – out	SMD: 0.41, 95%CI: 0.26 – 0.56, I^2 = 0%, P < 0.01

GDF-15, growth differentiation factor-15; IGF-1, insulin growth factor-1

Sensitivity analysis including only studies with lower risk of bias

Follistatin

Study or Subgroup	Sarcopenia Mean	SD	Total	No Sarcopenia Mean	SD	Total	Weight	Std. Mean Difference IV, Random, 95% CI	Year
Hofmann 2015	1.98	0.52	9	2.12	0.52	78	30.6%	-0.27 [-0.96, 0.42]	2015
Seo 2020	2.09	0.56	27	2.2	0.59	32	0.0%	-0.19 [-0.70, 0.33]	2020
Du 2021	18.97	6.1	51	13	4.9	193	36.6%	1.15 [0.83, 1.48]	2021
Echeverria 2021	3.19	1.23	15	2.68	1.28	59	32.8%	0.40 [-0.17, 0.97]	2021
**Total (95% CI)**			**75**			**330**	**100.0%**	**0.47 [-0.36, 1.30]**	

Heterogeneity: $Tau^2 = 0.47$; $Chi^2 = 15.53$, df = 2 (P = 0.0004); $I^2 = 87\%$
Test for overall effect: Z = 1.11 (P = 0.27)

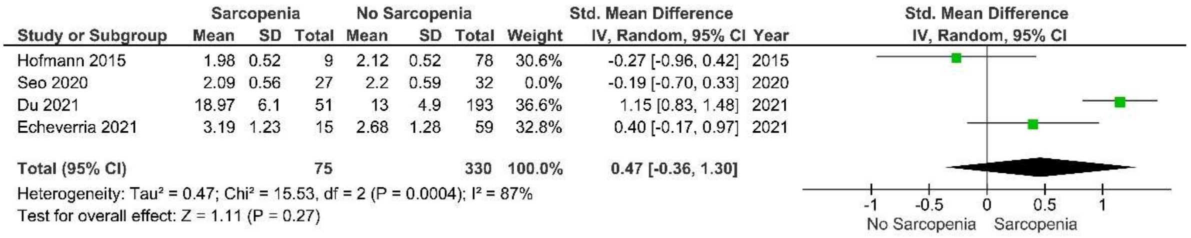

GDF-15

Study or Subgroup	Sarcopenia Mean	SD	Total	No Sarcopenia Mean	SD	Total	Weight	Std. Mean Difference IV, Random, 95% CI	Year
Hofmann 2015	1,522.2	524.6	9	1,393.2	530.8	78	11.2%	0.24 [-0.45, 0.93]	2015
Seo 2020	835.67	335.03	27	741.54	349	32	0.0%	0.27 [-0.24, 0.79]	2020
Nga 2021	1,542.3	288.1	21	1,478	139.9	104	18.3%	0.37 [-0.10, 0.84]	2021
Kim 2022	1,402.95	765	154	1,144.99	541.11	775	35.0%	0.44 [0.27, 0.62]	2022
Shin 2023	1,500	8,100	176	1,200	6,400	845	35.6%	0.04 [-0.12, 0.21]	2023
**Total (95% CI)**			**360**			**1802**	**100.0%**	**0.26 [-0.01, 0.54]**	

Heterogeneity: $Tau^2 = 0.05$; $Chi^2 = 10.99$, df = 3 (P = 0.01); $I^2 = 73\%$
Test for overall effect: Z = 1.92 (P = 0.06)

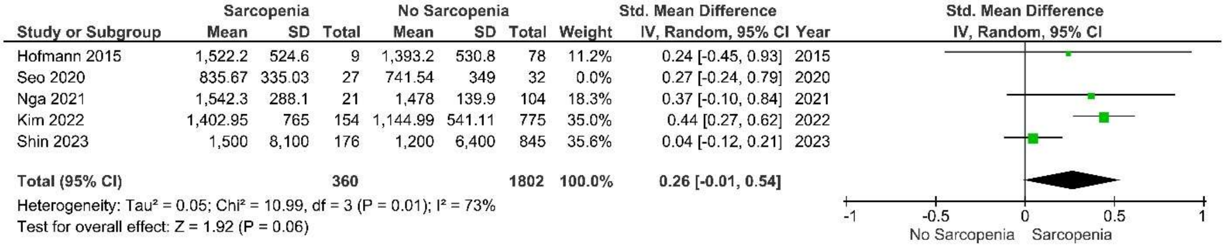

Growth hormone

Study or Subgroup	Sarcopenia Mean	SD	Total	No Sarcopenia Mean	SD	Total	Weight	Std. Mean Difference IV, Random, 95% CI	Year
Kuo 2018	0.47	0.66	50	0.35	0.45	681	47.9%	0.26 [-0.03, 0.54]	2018
Yee 2020	1.32	1.58	11	2.52	3	28	15.9%	-0.44 [-1.14, 0.27]	2020
Ferrari 2021	1.4	1.3	35	1.3	2.4	96	36.1%	0.05 [-0.34, 0.43]	2021
Xu 2022	0.3876	0.7083	57	0.2714	0.3888	64	0.0%	0.21 [-0.15, 0.56]	2022
**Total (95% CI)**			**96**			**805**	**100.0%**	**0.07 [-0.24, 0.38]**	

Heterogeneity: $Tau^2 = 0.03$; $Chi^2 = 3.41$, df = 2 (P = 0.18); $I^2 = 41\%$
Test for overall effect: Z = 0.44 (P = 0.66)

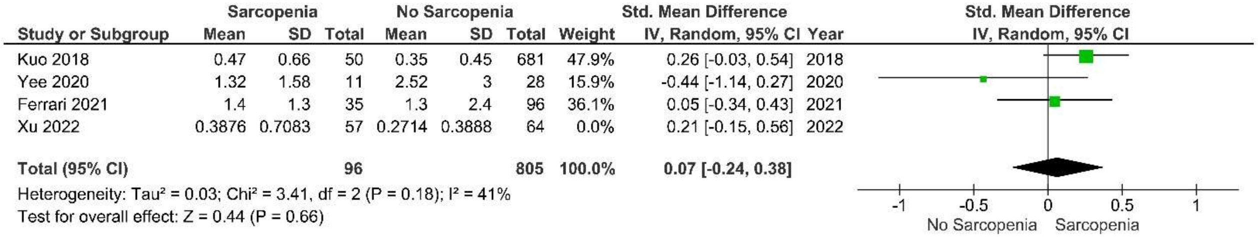

IGF-1

Study or Subgroup	Sarcopenia Mean	SD	Total	No Sarcopenia Mean	SD	Total	Weight	Std. Mean Difference IV, Random, 95% CI	Year
Volpato 2014	89.6	47.1	55	124.4	58.4	373	13.4%	-0.61 [-0.89, -0.32]	2014
Tay 2015	98.5	30.5	50	117.6	44	150	11.8%	-0.46 [-0.79, -0.14]	2015
Hofmann 2015	139.87	60.77	9	134.25	48.8	78	3.9%	0.11 [-0.58, 0.80]	2015
Li 2019	62.13	22.52	56	82.86	32.84	56	9.6%	-0.73 [-1.11, -0.35]	2019
Liang 2019	113.5	49.3	62	118.2	52.8	669	14.7%	-0.09 [-0.35, 0.17]	2019
Hata 2019	69	23.4	105	75.5	23.6	890	17.9%	-0.28 [-0.48, -0.07]	2019
Yee 2020	12.09	5.14	11	15.89	6.26	28	3.7%	-0.62 [-1.34, 0.09]	2020
Ferrari 2021	59.3	29.1	35	83.1	50.1	96	9.3%	-0.52 [-0.91, -0.13]	2021
Miyamoto 2021	71.5	24.5	39	85	30.8	453	11.6%	-0.44 [-0.77, -0.12]	2021
Xu 2022	111.37	38.57	57	131.99	45.7	64	0.0%	-0.48 [-0.84, -0.12]	2022
Lu 2022	1.02	0.72	60	1.12	0.77	9	3.8%	-0.14 [-0.84, 0.56]	2022
**Total (95% CI)**			**482**			**2802**	**100.0%**	**-0.39 [-0.54, -0.25]**	

Heterogeneity: Tau² = 0.02; Chi² = 15.23, df = 9 (P = 0.08); I² = 41%
Test for overall effect: Z = 5.20 (P < 0.00001)

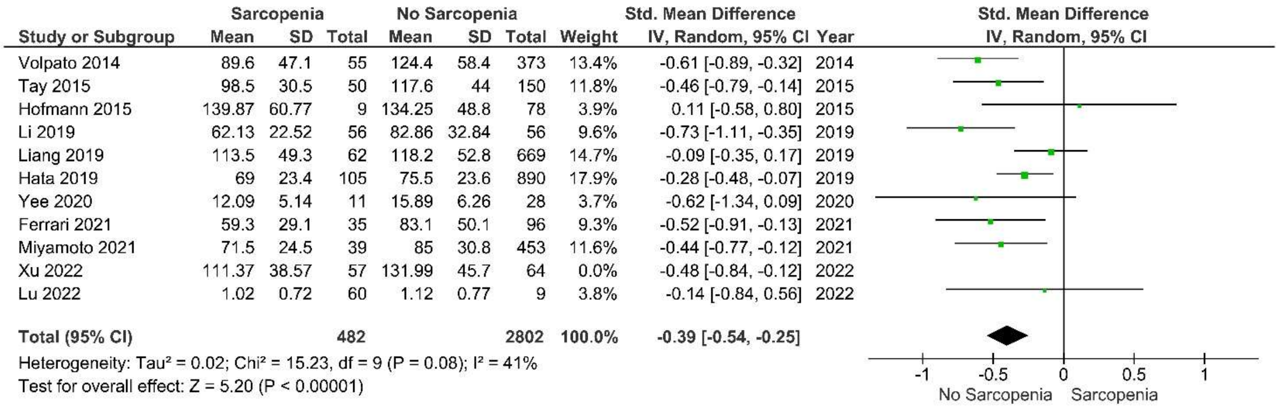

Myostatin

Study or Subgroup	Sarcopenia Mean	SD	Total	No Sarcopenia Mean	SD	Total	Weight	Std. Mean Difference IV, Random, 95% CI	Year
Hofmann 2015	2.43	1.41	9	3.12	2.14	78	9.5%	-0.33 [-1.02, 0.36]	2015
Tay 2015	28.6	8.4	50	27.5	7.6	150	15.9%	0.14 [-0.18, 0.46]	2015
Seo 2020	2.07	0.73	27	2.06	0.87	32	0.0%	0.01 [-0.50, 0.52]	2020
Du 2021	2.75	1.6	51	3.99	1.5	193	15.9%	-0.81 [-1.13, -0.50]	2021
Echeverria 2021	2.32	0.5	15	3.61	2.03	59	11.2%	-0.70 [-1.27, -0.12]	2021
Yen 2022	9.5	4.7	46	8.5	3.9	53	14.5%	0.23 [-0.17, 0.63]	2022
Shin 2023	3.2	1	176	3.5	1.2	845	18.4%	-0.26 [-0.42, -0.09]	2023
de Luis 2024	5.01	1.87	44	4.64	2.17	64	14.7%	0.18 [-0.21, 0.56]	2024
**Total (95% CI)**			**391**			**1442**	**100.0%**	**-0.20 [-0.50, 0.09]**	

Heterogeneity: Tau² = 0.12; Chi² = 30.28, df = 6 (P < 0.0001); I² = 80%
Test for overall effect: Z = 1.34 (P = 0.18)

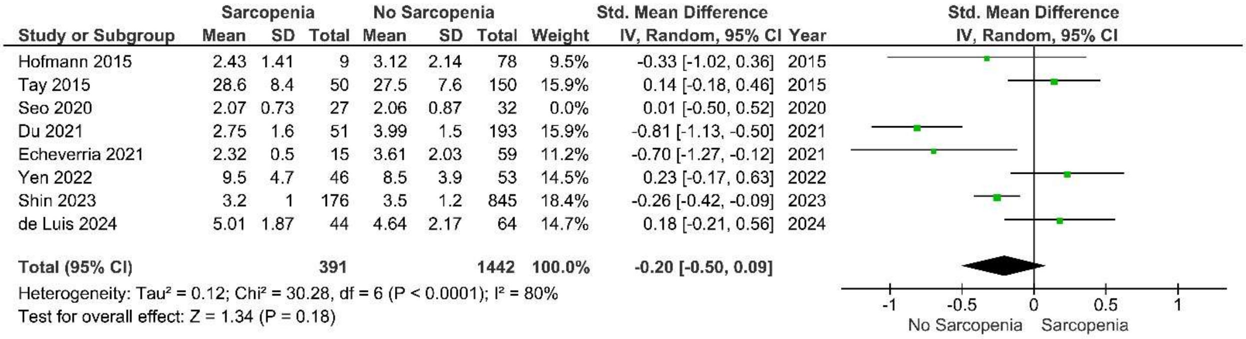

Table S1. Search terms employed in the screening based on title, abstract, and keywords in the literature search.

Database	Search terms
PubMed	(“sex hormone*” OR "total testosterone" OR "serum testosterone" OR "free testosterone" OR "bioavailable testosterone" OR androgen level* OR hypogonad* OR “IGF-1” OR “Insulin-like Growth Factor 1” OR “growth hormone” OR “activin A” OR “GDF15” OR “GDF-15” OR “Growth Differentiation Factor” OR “GDF-8” OR “GDF8” OR “myostatin” OR “FST” OR “follistatin”) AND sarcopeni*
Cochrane Library	(“sex hormone*” OR "total testosterone" OR "serum testosterone" OR "free testosterone" OR "bioavailable testosterone" OR androgen level* OR hypogonad* OR “IGF-1” OR “Insulin-like Growth Factor 1” OR “growth hormone” OR “activin A” OR “GDF15” OR “GDF-15” OR “Growth Differentiation Factor” OR “GDF-8” OR “GDF8” OR “myostatin” OR “FST” OR “follistatin”) AND sarcopeni*
Web of Science	(“sex hormone*” OR "total testosterone" OR "serum testosterone" OR "free testosterone" OR "bioavailable testosterone" OR androgen level* OR hypogonad* OR “IGF-1” OR “Insulin-like Growth Factor 1” OR “growth hormone” OR “activin A” OR “GDF15” OR “GDF-15” OR “Growth Differentiation Factor” OR “GDF-8” OR “GDF8” OR “myostatin” OR “FST” OR “follistatin”)

	AND sarcopeni*
Scopus	(“sex hormone*” OR "total testosterone" OR "serum testosterone" OR "free testosterone" OR "bioavailable testosterone" OR androgen level* OR hypogonad* OR “IGF-1” OR “Insulin-like Growth Factor 1” OR “growth hormone” OR “activin A” OR “GDF15” OR “GDF-15” OR “Growth Differentiation Factor” OR “GDF-8” OR “GDF8” OR “myostatin” OR “FST” OR “follistatin”) AND sarcopeni*

Table S2. Publication bias using Egger's test.

	p	t	b	95%CI
**IGF-1**	0.69	-0.4174	-0.3108	-0.7373 – 0.1157

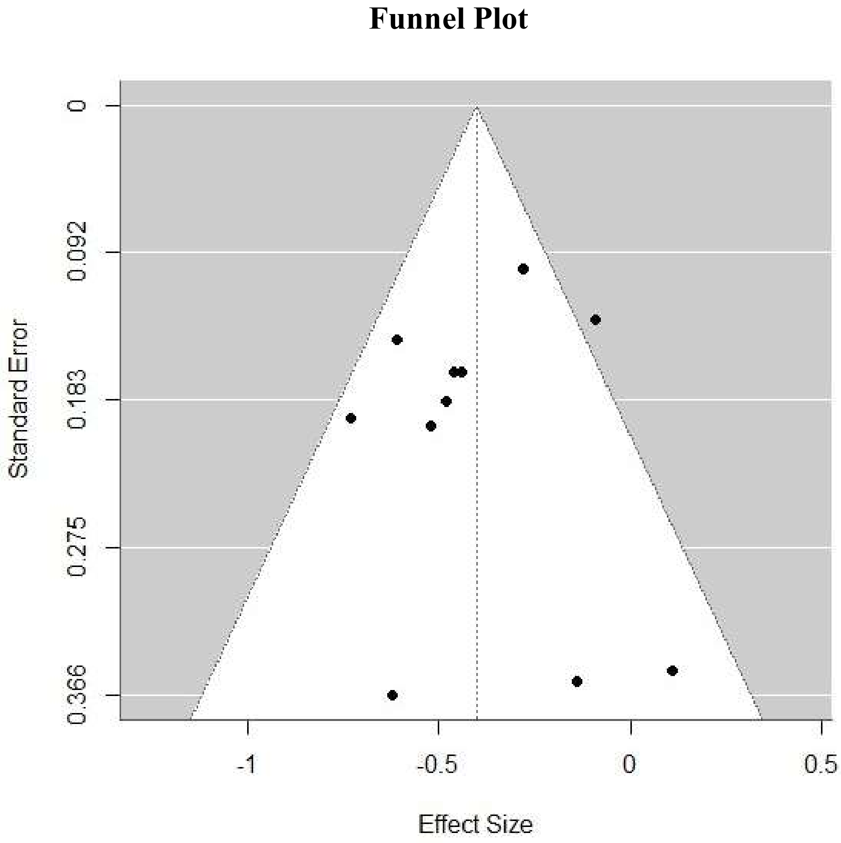

Table S3. Meta-regression analysis of serum IGF-1 levels differences between adults with and without sarcopenia.

**IGF-1**								
**Outcome**	**β**	**SE**	**z**	**p**	**95%CI**	**tau^2**	**I^2**	**R^2**
Age	-0.0286	0.0173	-1.6549	0.10	-0.06 – 0.01	0.0118	28%	37.78%
Body mass index	-0.1242	0.1302	-0.9543	0.34	-0.38 – 0.13	0.1403	42%	0%
Proportion of females	-0.0025	0.0048	-0.5150	0.61	-0.01 – 0.01	0.0198	38%	0%
Sarcopenia definition	-0.0377	0.0649	-0.5811	0.56	-0.16 – 0.09	0.0188	38%	0%
Body composition assessment tool	-0.0386	0.0724	-0.5329	0.59	-0.18 – 0.10	0.0208	39%	0%

CI, confidence interval; IGF-1, insulin growth factor-1.

Table S4. Risk of bias utilizing a modified version of the Newcastle Ottawa Scale for cross-sectional studies*

First Author	Representativeness of the sample	Sample size	Non-responders	Ascertainment of the exposure	Comparability based on the study design or analysis	Assessment of outcome	Statistical test	Total score
Aryana, 2019	*	-	*	**	**	*	*	8
Choi, 2024	*	-	*	**	**	*	*	8
De Luis, 2024	*	-	*	**	*	*	*	7
Diago-Galmes, 2021	*	-	-	*	**	*	-	5
Du, 2021	*	-	*	**	**	*	*	8
Echeverria, 2020	*	-	*	**	**	*	*	8
Ferrari, 2021	*	-	*	**	**	*	*	8
Hata, 2023	*	-	*	**	**	*	*	8
Hofmann, 2015	*	-	*	**	**	*	*	8
Ke, 2022	*	-	*	*	**	*	*	7
Kim, 2022	*	-	*	**	**	*	*	8
Kirk, 2020	*	-	*	**	**	*	*	8
Kuo, 2019	*	-	*	**	**	**	*	9
Li, 2019	_	*	*	**	**	*	*	8
Liang, 2023	*	-	*	**	**	*	*	8
Lu, 2022	*	-	*	**	**	*	*	8

First Author	Representativeness of the sample	Sample size	Non-responders	Ascertainment of the exposure	Comparability based on the study design or analysis	Assessment of outcome	Statistical test	Total score
Miyamoto, 2021	*	-	*	**	**	*	*	8
Nga, 2021	*	*	*	**	**	*	*	9
Seo, 2020	*	-	-	**	*	*	-	5
Shin, 2023	*	-	*	**	*	*	*	8
Surmeli, 2022	*	-	*	*	**	*	*	7
Tay, 2015	*	-	*	**	**	*	*	8
Volpato, 2014	*	-	*	**	**	*	*	8
Xu, 2022	-	-	*	**	*	*	-	5
Yee, 2020	-	-	*	**	*	*	*	6
Yen, 2022	-	-	*	**	**	*	*	7
